# MicroRNA-23b Inhibits the Proliferation and Migration of Heat-Denatured Fibroblasts by Targeting Smad3

**DOI:** 10.1371/journal.pone.0131867

**Published:** 2015-07-08

**Authors:** Xipeng Zhang, Jie Yang, Jiming Zhao, Pihong Zhang, Xiaoyuan Huang

**Affiliations:** 1 Department of Burns and Plastic Surgery, Xiangya Hospital, Central South University, Changsha, Hunan 410008, China; 2 Department of Pathology, Zhujiang Hospital, Southern Medical University, Guangzhou, Guangdong 510282, China; 3 Department of Traditional Chinese Medicine, Jinzhou Traditional Chinese Medicine Hospital, Zhangjiajie, Hunan 427000, China; Universitatsklinikum Freiburg, GERMANY

## Abstract

**Background:**

Skin grafting with the preservation of denatured dermis is a novel strategy for the treatment of burn-injured skin. Denatured dermis has the ability to restore to the morphology and function of normal skin, but the underlying molecular mechanism is elusive. MicroRNAs (miRNA) are small noncoding RNAs and regulate normal physiology as well as disease development. In this study, we assessed the potential role of miRNA-23b (miR-23b) in the regulation of cell proliferation and migration of heat-denatured fibroblasts and identified the underlying mechanism.

**Methods:**

The expression of miR-23b in denatured dermis and heat-denatured fibroblasts was detected by quantitative real-time polymerase chain reaction (RT-PCR). The effects of miR-23b on cell proliferation and migration of heat-denatured fibroblasts were assessed by transient transfection of miR-23b mimics and inhibitor. The target gene of miR-23b and the downstream pathway were further investigated.

**Results:**

miR-23b was downregulated in denatured dermis and heat-denatured fibroblasts. Downregulation of miR-23b dramatically promoted the proliferation and migration of heat-denatured fibroblasts. Subsequent analyses demonstrated that Smad3 was a direct and functional target of miR-23b in heat-denatured fibroblasts, which was validated by the dual luciferase reporter assay. Moreover, immunohistochemistry analysis showed that denatured dermis from rats displayed enhanced staining of Smad3. In addition, miR-23b modulated denatured dermis by activating the Notch1 and TGF-β signaling pathways.

**Conclusions:**

Our findings suggest that downregulation of miR-23b contributes to the recovery of denatured dermis, which may be valuable for treatment of skin burns.

## Introduction

Wound healing is a dynamic process characterized by various cellular and physiologic events, including acute and chronic inflammation, re-epithelialization and restoration of the underlying connective tissue. Denatured dermis resulting from deep skin burns is associated with disorder of cell metabolism, functional impairment and pathological changes in morphology, but can be reversed to the morphology and function of normal dermis [1]. Therefore, denatured dermis plays an essential role in wound healing and preservation of denatured dermis may be an effective treatment for skin burns to attenuate scar formation and restore normal appearance. It is widely accepted that large sheets of split-thickness autologous skin grafting with the preservation of denatured dermis may be the optimal choice for the management of deep partial-thickness burns [2]. Animal studies have shown that the thickness, structure and morphology of the grafted skin are similar to that of normal tissues 21 days following grafting [3]. Furthermore, autologous skin grafting on deep partial-thickness burns, where the depth of retained denatured dermis is 0.10 mm, may help regenerate dermal function and alleviate scar formation [4]. However, the mechanisms by which denatured dermis participate in remodeling the structure in the latter stage of wound healing remain unclear.

MicroRNAs (miRNAs) are small noncoding RNAs and inhibit target gene expression at the post-transcriptional level [5]. miRNAs have been demonstrated to act through binding to the 3’ untranslated region (3’UTR) of target gene mRNA, resulting in inhibition of protein translation or cleavage of targeted mRNA [6,7]. miRNAs are involved in the regulation of most cellular processes, including cell proliferation, apoptosis, differentiation, the timing of developmental transition and organ development [8]. Accumulating evidence has demonstrated that deregulation of miRNAs is associated with the pathogenesis of many common human diseases, such as cancer. Moreover, miRNAs are involved in the regulation of embryogenesis, during which multipotent progenitors within the single-layered surface epithelium differentiate to form the epidermis [9]. Recently, numerous studies have shown that miRNAs are differentially expressed in the different phases of wound healing, suggesting pivotal roles of miRNAs during the multistep processes of wound healing. For example, miR-105, miR-125b and miR-140 are involved in the inflammatory phase; miR-15a, miR-15b and miR-16 participate in the granulation phase; and miR-29 and miR-192 function in the remodeling phase [10]. In addition, miRNAs are involved in different phases in wound healing impairment in diabetes by regulating cell re-epithelialization, migration, angiogenesis and differentiation [11]. Very interestingly, a recent miRNA microarray analysis between denatured dermis and normal skin of patients revealed distinct expression profiles of mRNAs, in which miR-23b was found to be significantly downregulated in denatured dermis [12]. However, the molecular mechanism and physiologic functions of the downregulation of miR-23b in the recovery of denatured dermis during wound healing is unknown. In this study, the roles of miR-23b in denatured dermis were explored. The expression of miR-23b during the recovery of denatured dermis was evaluated. Furthermore, the roles of miR-23b in denatured dermis and the underlying mechanisms were investigated.

## Material and Methods

### Establishment of rat burn models

Male Sprague-Dawley (SD) rats with weight of 220-250g were purchased from the Central South University Department of Experimental Animals (Changsha, China). Rats were housed in a climate-controlled room under a 12:12-h light-dark cycle, with free access to food and water. A deep partial-thickness burn model in SD rats was established in accordance with the United States Public Health Service Policy on Humane Care and Use of Laboratory Animals and the Guide for the Care and Use of Laboratory Animals (1996). First, rats were anesthetized with 10% chloralhydrate (0.5 mL/100 g). The backs of the rats were completely shaved with an electrical clipper. Second, an aluminum cylinder (3.76 cm in diameter, 3.78 cm in height) was placed into 75°C water for 10 min and pressed on the back of rats for 10 s to produce a deep partial-thickness burn wound, which was confirmed by pathological examination. The effective wound diameter was 2.5 cm. Denatured dermis was harvested following anesthetizing rats with 10% overdose chloralhydrate (0.5 mL/100 g) at days 1, 3, 5 and 7 after burn creation and then rats were killed by decapitation. Normal skin samples were also collected from the back of the rats. The isolated skin tissues were immediately frozen in liquid nitrogen and stored at -80°C for further analyses. A control group did not receive any treatment. This study was reviewed and approved by the Ethics Committee of The Central South University. All efforts were made to minimize animal suffering and the number of animals used.

### Primary culture and treatment of human dermal fibroblasts

Normal human skin was obtained from the remaining skin in plastic surgery procedures after obtaining informed consent from the patients. The skin sample was minced in tissue culture medium with a surgical scalpel and subsequently digested by trypsin. Following brief centrifugation, the supernatant containing the isolated cells were cultured in Eagle’s minimal essential medium (EMEM) supplemented with 10% fetal bovine serum (FBS) at 37°C in a humidified atmosphere of 5% CO_2_ until reaching confluency. Subsequently, adherent fibroblasts were reseeded on new culture plates with EMEM containing 10% FBS, 100 IU/mL penicillin and 0.1 mg/mL streptomycin. Heat injury of fibroblasts was carried out by placing the cell culture plates in a water bath at 52°C for 30 s. In the control group, cell culture plates with fibroblasts were put in a water bath at 37°C for 30 s [13].

### Transfection

The miR-23b mimics, mimics negative control (mimics control), inhibitor, inhibitor negative control (inhibitor control), the small interfering RNA targeting Smad3 (siSmad3) and control-siRNA (siCtrl), the Smad3 overexpression plasmid (pcDNA-Smad3) and negative control (Ctrl) were purchased from Genepharma Inc (Shanghai, China). Cells were seeded at a density of 2–3×10^5^ cells per well in six-well plates for 24 h. Cells were then transfected with oligonucleotides or plasmid, using Lipofectamine 2000 (Invitrogen) following the manufacturer’s instructions. Cells that were treated with blank Lipofectamine 2000 alone were denoted the Mock control group. Cells were harvested at the indicated time for cell proliferation assays, protein analysis and migration assays.

### Protein extraction and Western blot analysis

Total protein from cells was extracted with RIPA Lysis Buffer (Cell Signaling Technology, CST). Ten micrograms of protein were separated by 10% sodium dodecyl sulfate—polyacrylamide gel electrophoresis and transferred onto polyvinylidenefluoride (PVDF) membranes. After blocking for nonspecific binding with 5% w/v non-fat dry milk dissolved in Tris buffered saline plus Tween-20 (TBS-T; 0.1% Tween-20; pH 8.3) at room temperature for 1 h, blots were incubated with antibodies against Smad3 (1:1000 dilution, CST), Notch1(1:1000 dilution, CST), TGF-β (1:1000 dilution, CST), β-actin (1:5000 dilution, Sigma) at 4°C overnight. After incubation with secondary antibody conjugated to horseradish peroxidase at room temperature for 1 h, protein bands were visualized using enhanced chemiluminescence detection kit (GE Healthcare, Waukesha, WI, USA). Specific protein signal was normalized to β-actin. All of the experiments were performed in triplicate.

### RNA extraction and real-time quantitative polymerase chain reaction

Total RNA was extracted using TRIzol Reagent (Invitrogen, CA, USA). Five hundred ng of total RNA was used for cDNA synthesis using a High-capacity RNA-to-cDNA Kit (Applied Biosystems, Foster City, CA). miScript Reverse Transcription Kit (Qiagen, Hilden, Germany) was used for reverse transcription of miRNAs. Quantitative real-time polymerase chain reaction (PCR) was carried out in ABI Prism 7500 Sequence Detection System (Applied Biosystems) using SYBR Premix Ex Taq (TaKaRa, Dalian, China) according to the manufacturer’s instructions. The miRNA sequence-specific reverse transcription (RT)-PCR for miR-23b and endogenous control U6 was performed using Hairpin-it miRNAs qPCR quantitation kit and U6 snRNA real-time PCR normalization kit (GenePharma, Shanghai, China). All reactions including no-template controls were performed in triplicate.

### Cell proliferation assay

Cell proliferation was measured using the Cell Counting Kit-8 (CCK-8; Dojindo Corporation, Kumamoto, Japan). The principle of the kit relies on the phenomenon that in the presence of electron coupling reagent, WST-8 can be deoxidated by dehydrogenase in mitochondria and generate a highly water-soluble orange product (formazan). The color depth generated is proportional to the cell proliferation. Fibroblasts were seeded into 96-well plates at a density of 1 ×10^5^ cells/well in 200 μl medium. 10μl of CCK-8 solution were added to 90 μl of culture medium following treatment at the indicated time and incubated at 37°C for 2 h. The optical density was measured at 450 nm. Each group included at least 6 replicate wells, and all of the experiments were performed in triplicate.

### In vitro scratch and Transwell migration assays

Scratch and Transwell migration assays were performed to evaluate cell migration. When reaching 90% confluence, cells were scratched with a 100 μl pipette tip and cultured in serum-free medium for 24 h and 48 h to recover from the disruption. For the Transwell migration assay, cells were plated on the upper surface of the Transwell chamber (Corning, New York, NY, USA) and incubated for 24 h. Cells that migrated into the lower surface of the chamber were fixed with 75% methanol and stained with crystal violet (Sigma). Five fields were randomly selected from each experiment to quantify migrated cells under a microscope. All of the experiments were performed in triplicate.

### Dual luciferase reporter assay

The 3’untranslated region (3’UTR) of Smad3 containing the miR-23b binding sites was amplified by PCR from human genomic DNA. The wild-type 3’UTR of Smad3 as well as mutant 3’UTR with nucleotide substitutions in the putative binding sites corresponding to the seed sequence of miR-23b were inserted into the psiCHECK-2 vector immediately downstream of the stop codon of luciferase to develop psiCHECK2-Smad3-3’UTR and psiCHECK-Smad3-mut-3’UTR, respectively. Either of these vectors was co-transfected with miRNAs into cells using Lipofectamine 2000 according to the manufacturer’s protocols. Luciferase activity was measured 48 h after transfection using the Dual Luciferase Reporter Assay Kit (Promega). Three independent experiments were performed and the data are presented as the mean ± standard deviation (SD).

### Immunohistochemical staining

Denatured dermis from rats was fixed with formalin, embedded in paraffin blocks and cut into series of sections (5 μm). Paraffin-fixed tissue sections were deparaffinized twice with xylol for 15 min, and rehydrated with graded alcohol. After blocking endogenous peroxidase with 0.3% hydrogen peroxide and goat serum, the slides were subjected to antigen retrieval by heating sections in 10 mM citrate buffer (pH 6.0). The slides were then incubated with a primary antibody against Smad3 (1:100 dilution, CST) at 4°C overnight. The next day, after washing with PBS, the sections were incubated with biotinylated secondary antibodies (Amersham). Incubation of avidin biotin complex was carried out according to the Vector Elite ABC detection kit (Vector Labs, Burlingame, USA). Tissue sections were counterstained with hematoxylin, and dehydrated in an ascending series of ethanol (85–100%). After xylol treatment, sections were mounted. Brown-yellow granules in the cytoplasm were considered positive staining.

### Statistical analysis

The data are presented as mean ± SD. Each experiment was repeated at least three times. The two-tailed Student’s t-test was used to evaluate the statistical significance of the difference between two groups. Statistical comparisons of more than two groups were performed using one-way analysis of variance (ANOVA). SPSS 16.0 (IBM) software was used for all statistical analysis. A P value of < 0.05 was considered statistically significant.

## Results

### Expression of miR-23b in normal skin and heat-denatured dermis

**Fig 1:**
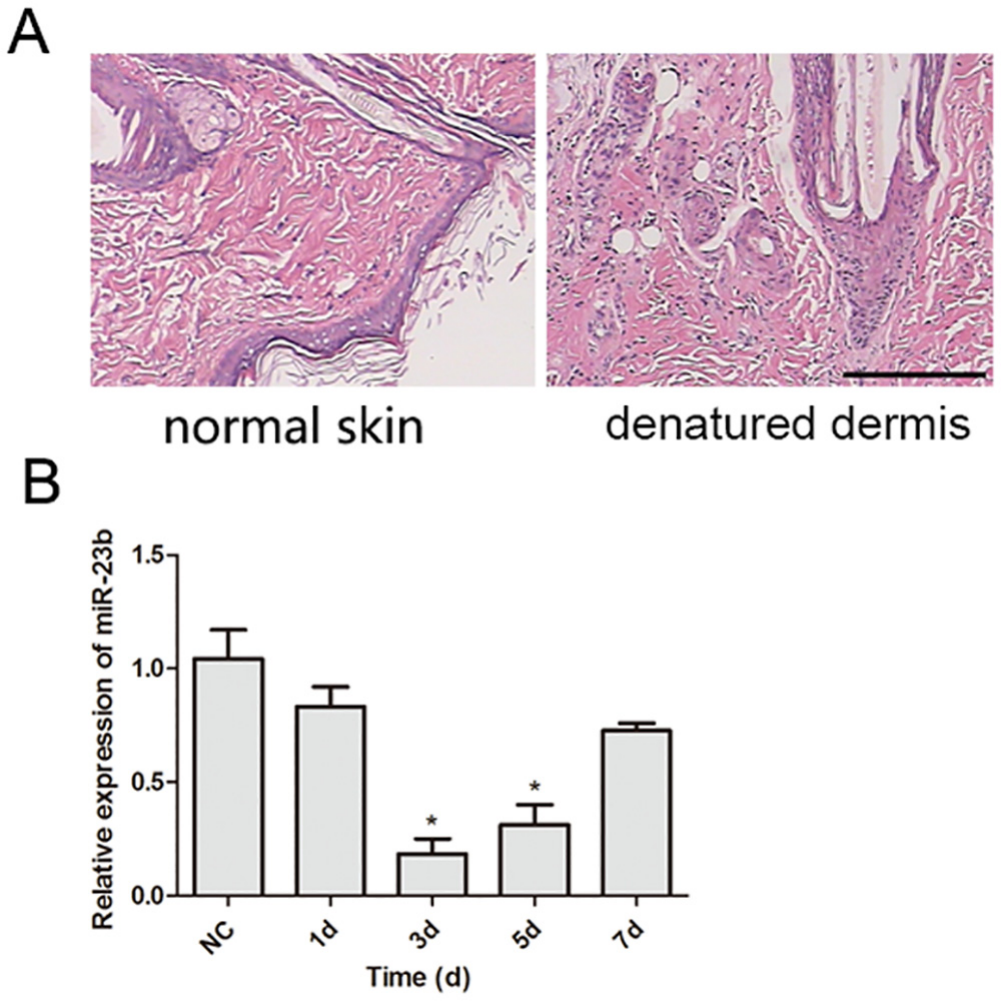
Histological examination of denatured dermis and the expression of miR-23b in denatured dermis. (A) HE staining of denatured dermis 3 days following heat damage revealed nucleus pyknosis of the epidermal and follicular epithelial cells, disintegration of the sebaceous gland, and swelling and fusion of collagen compared to normal skin. (B) The relative expression level of miR-23b was examined by quantitative RT-PCR in normal rat skin (NC), and denatured dermis at different recovery times (1, 3, 5, 7 days) after burn creation. Data are shown as mean ± SD derived from three independent experiments. **, P<0.01, versus NC.

To explore the expression of miR-23b during the recovery of denatured dermis, the skin tissues of the deep partial-thickness burn rats were collected at different time points after burn creation and stained with hematoxylin and eosin (HE). Three days following burn creation, skin tissues showed pathological characteristics of a deep partial-thickness burn wound including nucleus pyknosis of the epidermal and follicular epithelial cells, disintegration of the sebaceous gland, and swelling and fusion of collagen (Fig 1A). Next, the expression of miR-23b in the denatured dermis was measured at different recovery time points after burn creation (1, 3, 5, and 7 days) by RT-PCR. As shown in Fig 1B, miR-23b expression demonstrated a dynamic alteration during the recovery of denatured dermis. Compared with that of normal skin, miR-23b expression significantly decreased in denatured dermis, with the greatest inhibitory rate of 81% after three days, but then gradually increased three days post-burn.

### Expression of miR-23b in heat-denatured human fibroblasts

**Fig 2:**
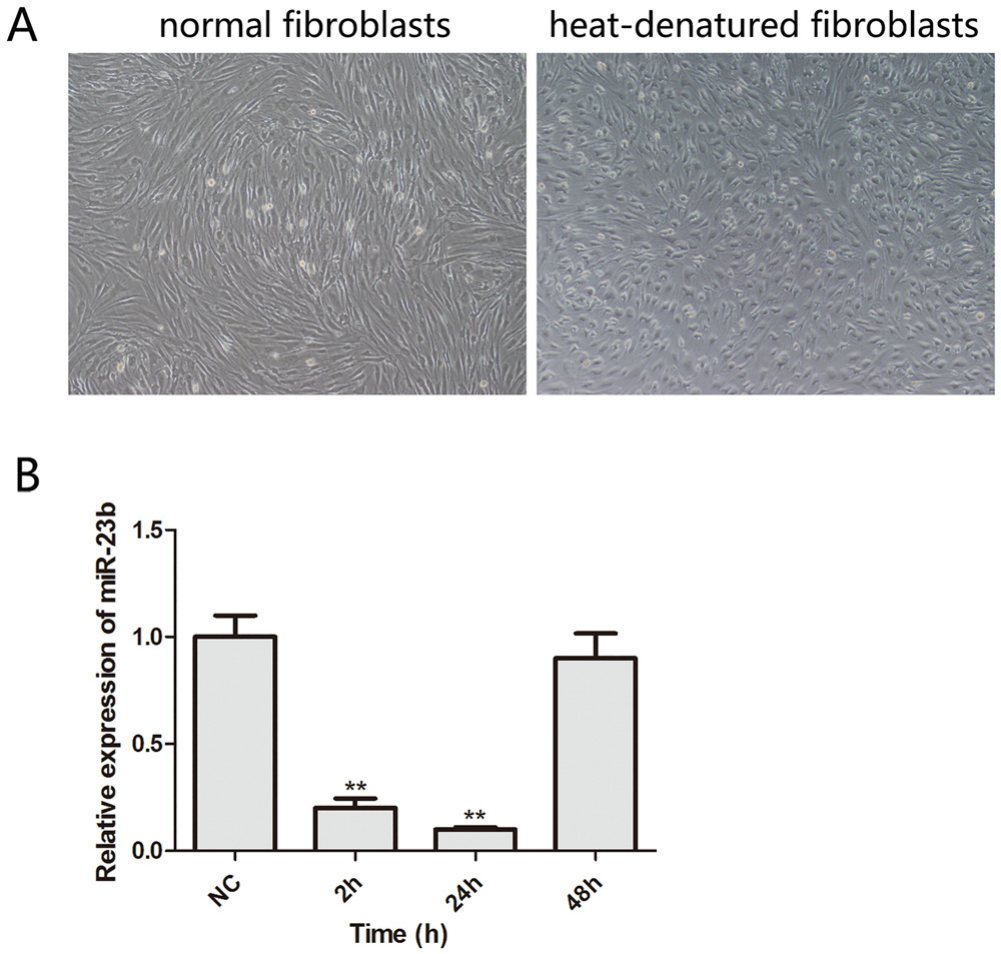
Morphological change of heat-denatured fibroblasts and the expression level of miR-23b in heat-denatured fibroblasts. (A) Fibroblasts were subjected to heat damage. After 6 h, cell morphology was observed under a light microscope. Scale bar: 200 μm. (B) The expression of miR-23b was examined by quantitative RT-PCR in fibroblasts at different times (1, 3, 5 days) after heat damage. **P < 0.01, versus NC.

To explore the physiologic functions of miR-23b during the recovery of denatured dermis, a model of human heat-denatured fibroblasts was established following the methods described by Li et al. [13]. As expected, in comparison to untreated fibroblasts, heat-denatured fibroblasts showed sunken cytomembrane and transparent vacuoles in the cytoplasm six hours after heat damage (Fig 2A). The miR-23b levels in heat-denatured fibroblasts at different time points following heat damage were determined by RT-PCR. Similar to denatured dermis, heat-denatured fibroblasts also demonstrated a time-dependent change in miR-23b expression. Compared with that in normal fibroblasts, miR-23b significantly decreased to 20% after 2h and 10% after 24h, while dramatically increased to the levels of normal fibroblasts two days after heat damage. Taken together, our results clearly demonstrate that miR-23b is dynamically regulated during the recovery of both denatured dermis and heat-denatured human fibroblasts, suggesting an essential role for miR-23b during wound healing.

### Downregulation of miR-23b promotes the proliferation and migration of heat-denatured fibroblasts

**Fig 3:**
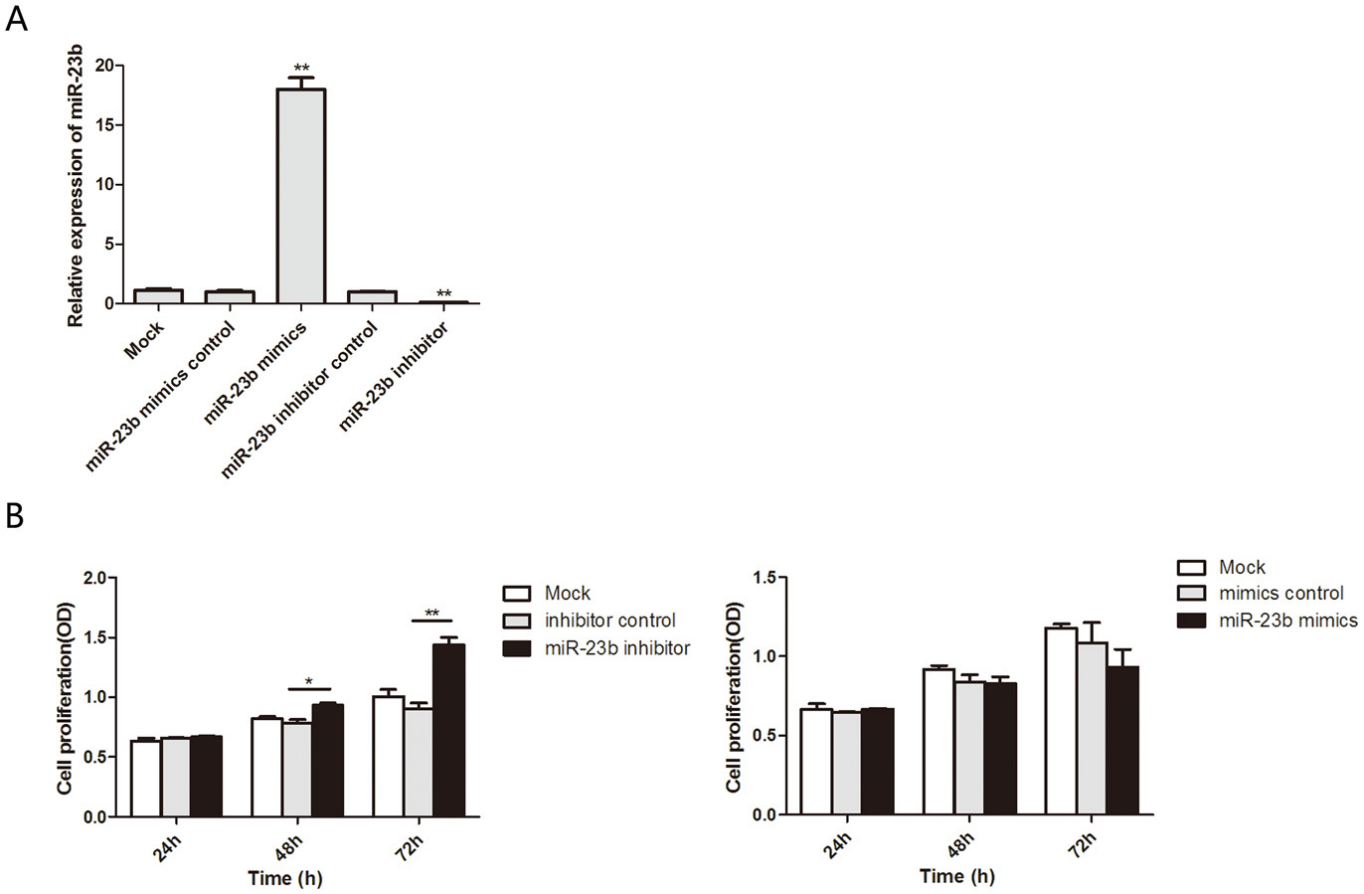
MiR-23b suppressed the proliferation of heat-denatured fibroblasts. (A) miR-23b expression level was examined by quantitative RT-PCR after transfection with mimics and inhibitor. (B) Cell proliferation was measured by CCK-8 assay after treatment with the miR-23b mimics, inhibitor or empty vector. *P < 0.05, **P < 0.01, mimics versus control, inhibitor versus control.

**Fig 4:**
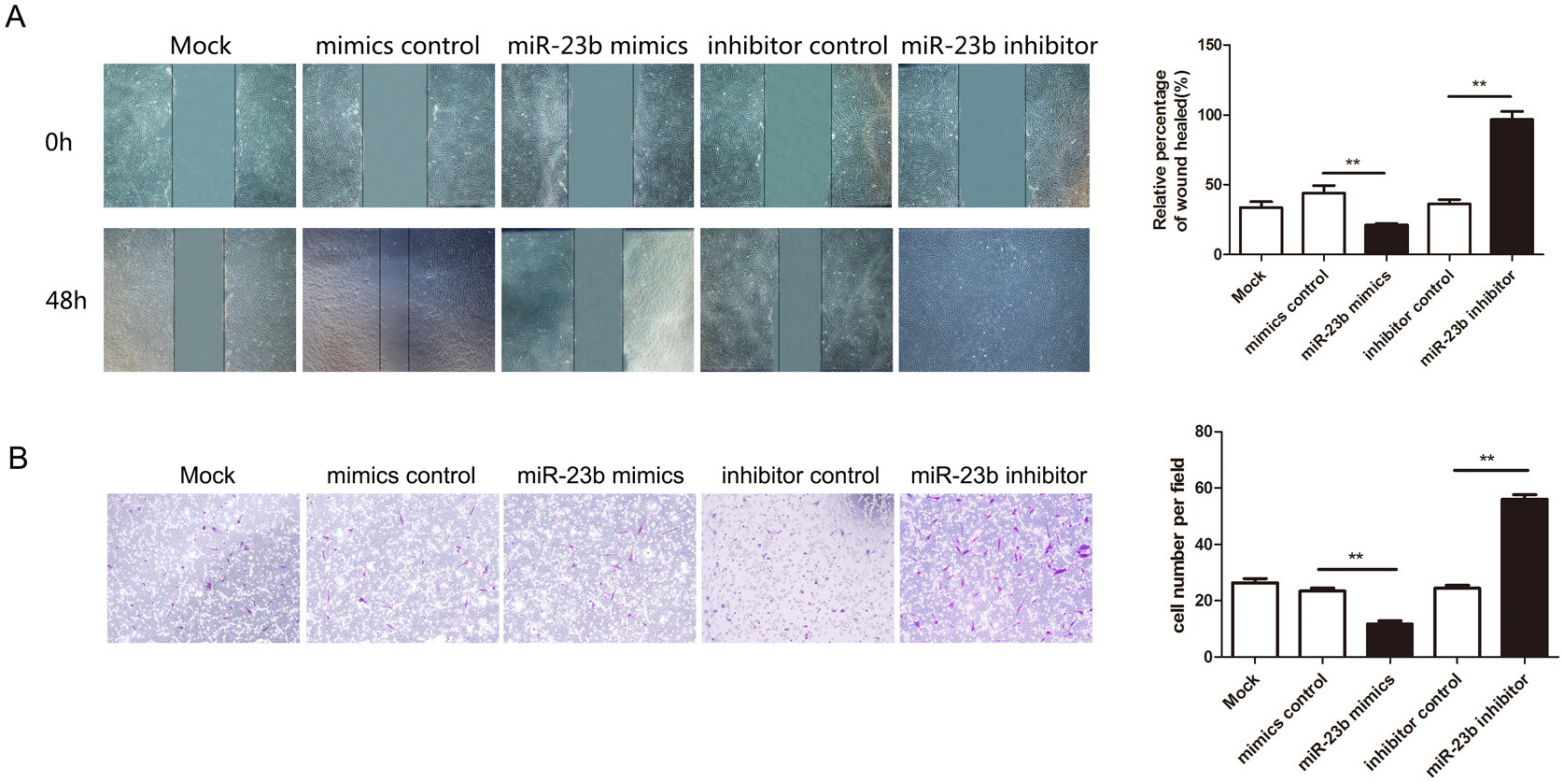
MiR-23b suppressed migration of heat-denatured fibroblasts. (A) Cell migration was quantified by scratch assay. Cells were imaged 0 h and 48 h after scratches were created. (B) Transwell cell migration assay. Representative images of cells that had migrated into the lower chamber are shown. *P < 0.05, **P < 0.01, mimics versus control, inhibitor versus control.

To investigate the biological function of miR-23b in heat-denatured fibroblasts, human fibroblasts were transfected with miR-23b mimics to increase the endogenous level of miR-23b, or miR-23b inhibitor to decrease the level of miR-23b. qRT-PCR was performed to validate the level of miR-23b after transfection. Compared with miR-23b mimics and inhibitor negative controls, transfection of miR-23b mimics led to a dramatic increase of miR-23b, and transfection of miR-23b inhibitor suppressed endogenous miR-23b expression (Fig 3A). Next, fibroblasts were subjected to heat damage 12 h after transfection of miR-23b mimics or inhibitor and cell proliferation was assessed by CCK-8 assay at 24, 48 and 72 h after transfection. After 48 h, the proliferation of cells transfected with miR-23b inhibitor significantly increased compared with cells transfected with scramble mimics or untreated cells. However, there was no significant difference in the viability of cells transfected with miR-23b mimics compared with control cells (Fig 3B). To investigate whether miR-23b regulates fibroblast migration following heat damage, in vitro scratch and Transwell migration assays were performed on heat-denatured fibroblasts following miR-23b transfection. Results showed that the migration capacity of cells transfected with miR-23b inhibitor was dramatically increased compared with the scramble and control group and the migration capacity of cells transfected with miR-23b mimics was dramatically decreased compared with the scramble and control group cells (Fig 4). These results clearly demonstrate that suppression of miR-23 promotes the proliferation and migration of heat-denatured fibroblasts, suggesting that downregulation of miR-23b might be required for the recovery of heat-denatured dermis and fibroblasts.

### MiR-23 directly targets Smad3

**Fig 5:**
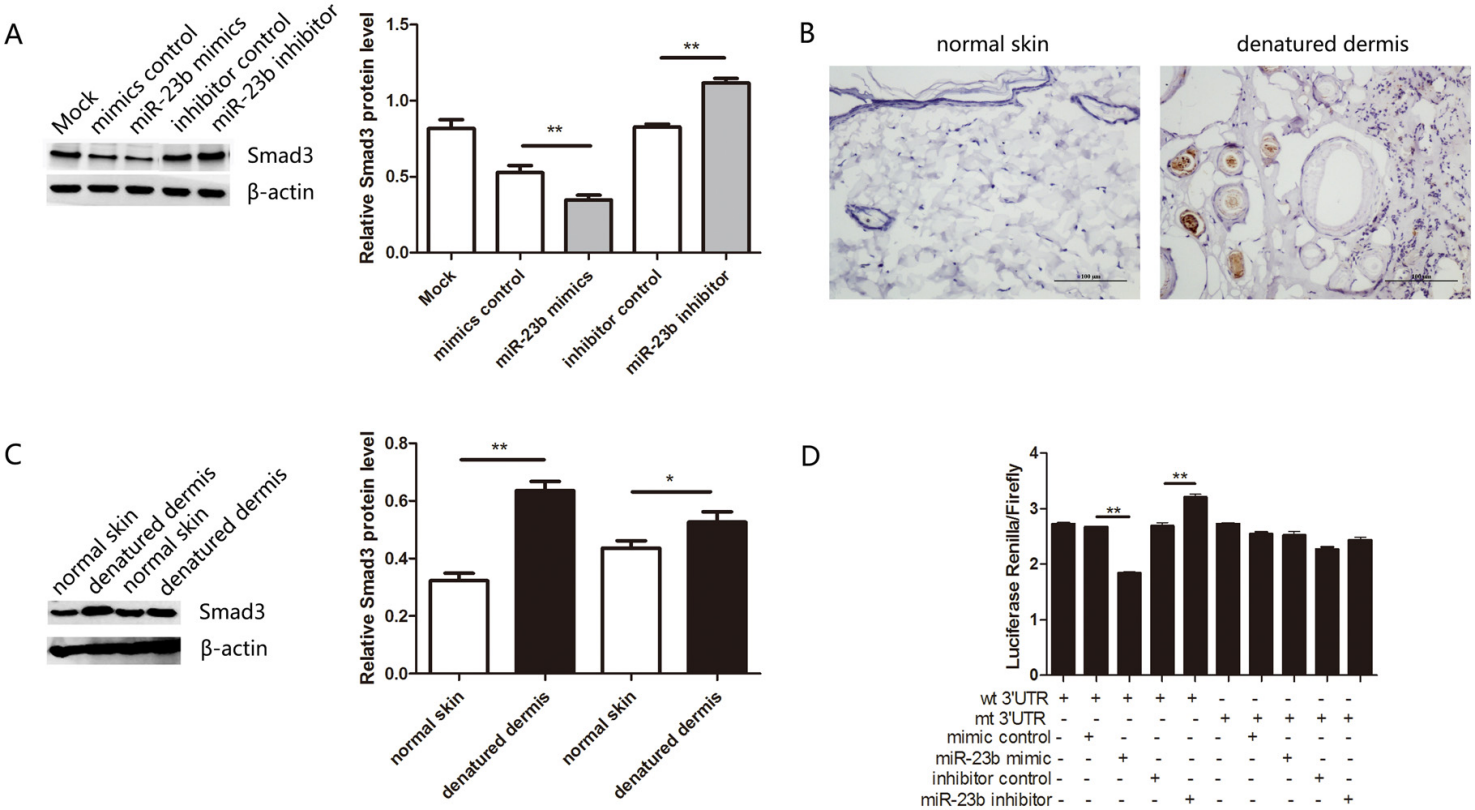
Smad3 is a direct target of miR-23b. (A) Western blotting analysis of Smad3 expression after transfection of miR-23b mimics, inhibitor and control in fibroblasts. (B) The intensity of Smad3 staining in denatured dermis of rats was examined by immunohistochemistry three days after burning. Brown-yellow granules in nucleus or cytoplasm were considered positive staining. Scale bar: 100um. (C) Western blotting analysis of Smad3 expression in normal skin and denatured dermis of rats. (D) Dual luciferase assays showed an increase after transfection of miR-23b inhibitor or a decrease after transfection of miR-23b mimics. *P < 0.05, **P < 0.01, mimics versus control, inhibitor versus control, and denatured dermis versus normal skin.

To well underlying molecular mechanism of miR-23b suppresses the proliferation and migration of heat-denatured fibroblasts, bioinformatic analysis of three publicly available algorithms (PicTar, TargetScan and miRBase) was performed to explore the target gene of miR-23b. Results revealed complementarity between Smad3 3’UTR and miR-23b. To test whether Smad3 can be regulated by miR-23b, the protein level of Smad3 in heat-denatured human fibroblasts transfected with miR-23b mimics or inhibitor was determined by Western blotting. The results showed that miR-23b mimics dramatically decreased the protein level of Smad3, while miR-23b inhibitor increased the Smad3 protein level in heat-denatured human fibroblasts (Fig 5A). Furthermore, in agreement with the decreased expression of miR-23b (Fig 1B), immunohistochemistry of denatured dermis showed that the staining of Smad3 in denatured dermis three days after burning was much stronger than that in normal skin (Fig 5B). In addition, immunoblotting also revealed a marked increase of Smad3 in denatured dermis compared to normal skin from rats (Fig 5C).

To validate that Smad3 is a direct target of miR-23b, Smad3 luciferase reporters were constructed with wild-type and mutated 3’UTR of Smad3. These reporters were co-transfected with miR-23b mimics or inhibitor into heat-denatured human fibroblasts. Luciferase activity was measured by dual luciferase assays. As shown in Fig 5D, the luciferase activity was significantly decreased or increased compared to either mutant or empty controls when co-transfected with miR-23b mimics or inhibitor, respectively. These results demonstrate that miR-23b binds directly to the 3’UTR of Smad3 to repress its expression in heat-denatured fibroblasts.

### Smad3 promotes the proliferation and migration of heat-denatured fibroblasts

**Fig 6:**
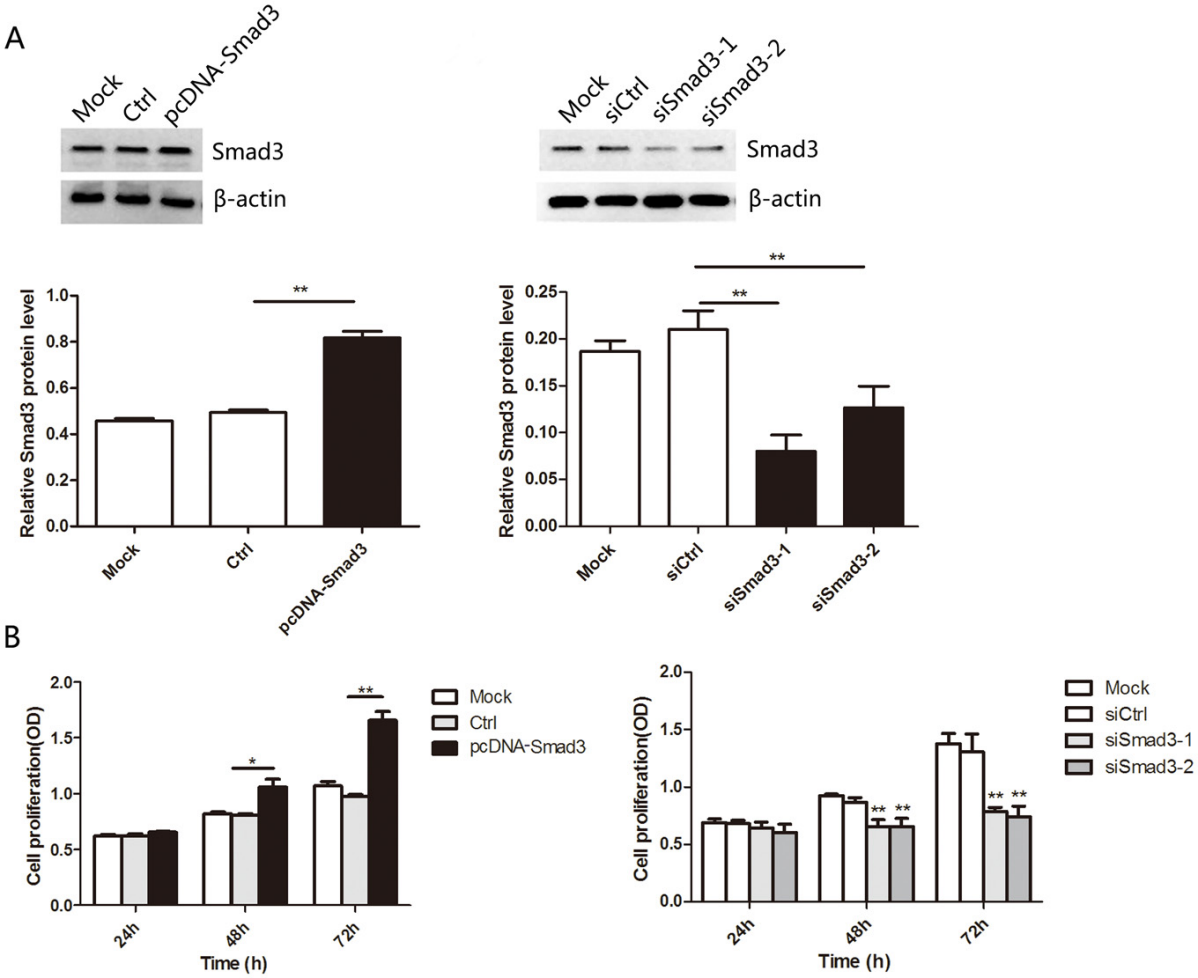
Smad3 promoted the proliferation of heat-denatured fibroblasts. (A) Western blotting analyses confirmed upregulation and downregulation of Smad3 in fibroblasts by pcDNA-Smad3 plasmid or siRNA, respectively. (B) Cell proliferation was measured by CCK-8 assay after transfection with pcDNA-Smad3 or siRNA. *P < 0.05, **P < 0.01, siRNA group versus control group, pcDNA group versus control group.

**Fig 7:**
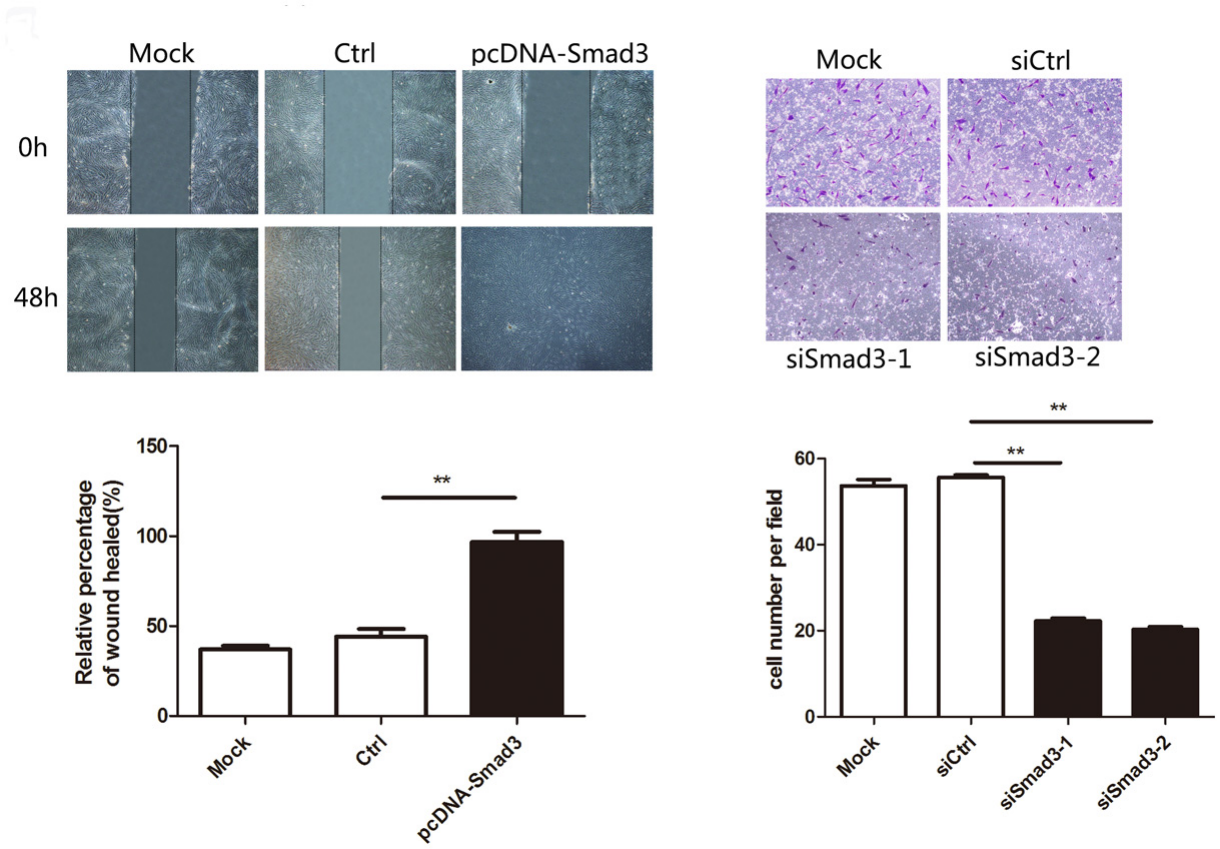
Smad3 promoted migration of heat-denatured fibroblasts. The migratory properties of cells transfected with pcDNA-Smad3 or siRNA were analyzed using scratch assay or Transwell migration assay. *P < 0.05, **P < 0.01, siRNA group versus control group, pcDNA group versus control group.

Our above observations suggest that upregulation of Smad3 might be required for the recovery of heat-denatured dermis and fibroblasts. To test this hypothesis, we assessed the cell growth and the migratory capability of heat-denatured fibroblasts after up or down-regulation of Smad3. Western blotting was performed to validate the level of Smad3 in fibroblasts after transfection with plasmid or siRNA (Fig 6A). The CCK-8 proliferation assay showed that the growth rate was increased in fibroblasts transfected with pcDNA-Smad3 compared with cells transfected with control. Meanwhile, the growth rate was reduced while transfecting with Smad3 siRNA compared with control. (Fig 6B). Moreover, the migratory capacity of cells transfected with pcDNA-Smad3 was dramatically increased compared with the control group and the migratory capacity of cells transfected with siRNA was decreased compared with the control group cells. (Fig 7). These results suggested that up-and down-regulation of Smad3 mimicked the effect of decreasing and increasing miR-23b expression in heat-denatured fibroblasts.

.

### Modulation of the Notch1 and TGF-β signaling pathways by miR-23b and Smad3

**Fig 8:**
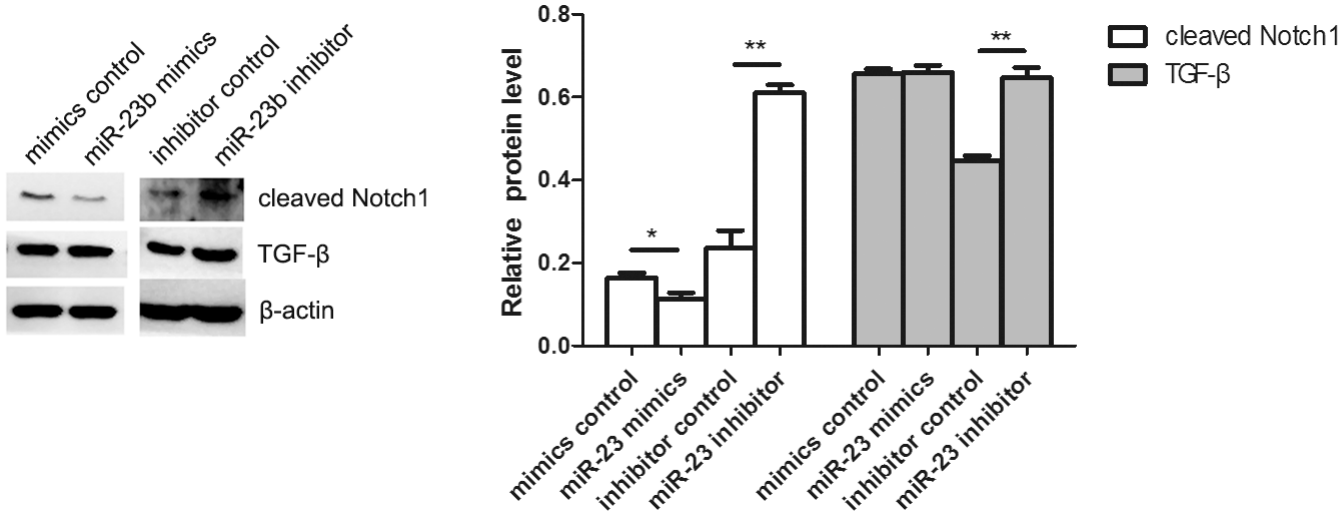
Regulation of the Notch1 and TGF-β signaling pathways by miR-23b. Western blotting analysis of cleaved Notch1 and TGF-β expression in fibroblasts after transfection with miR-23b mimics or inhibitor. *P < 0.05, **P < 0.01, miR-23b mimics versus control, inhibitor versus control.

**Fig 9:**
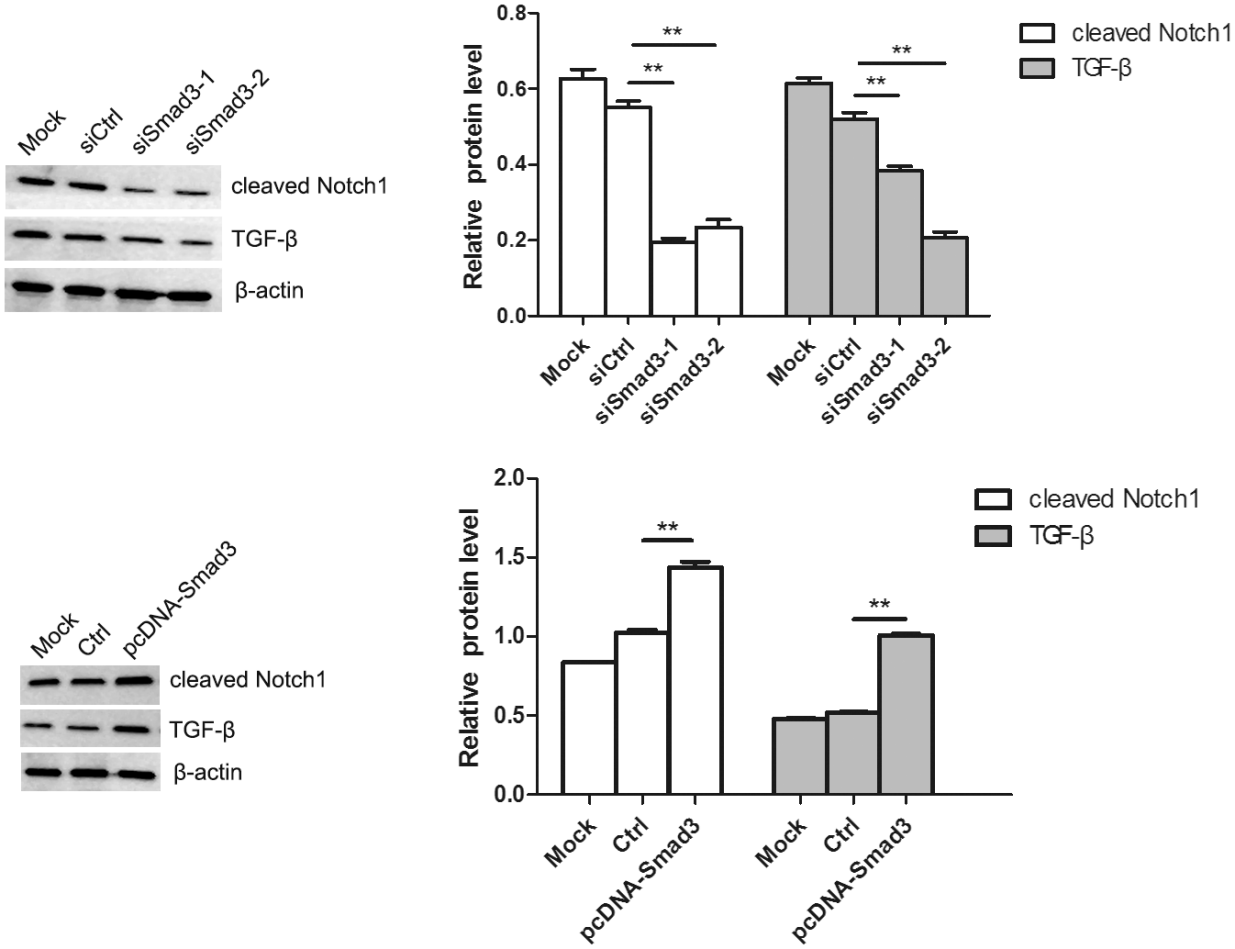
Regulation of the Notch1 and TGF-β signaling pathways by Smad3. Western blotting analysis of cleaved Notch1 and TGF-β expression after transfection with siRNA or plasmid of Smad3. *P < 0.05, **P < 0.01, siRNA group versus control group, pcDNA group versus control group.

To test whether the Notch1 and TGF-β signaling pathways are involved in miR-23b-modulated fibroblasts growth and migration after heat damage, the protein levels of TGF-β and cleaved Notch1 in heat-denatured human fibroblasts transfected with miR-23b mimics or inhibitor were determined by Western blotting. It was found that transfection with miR-23b inhibitor increased the levels of Notch1 and TGF-β, while miR-23b mimics decreased the expression of Notch1, but not TGF-β (Fig 8A). In addition, upregulation of Smad3 by transfection of plasmid in fibroblasts resulted in increased protein expression of Notch1 and TGF-β, whereas downregulation of Smad3 by transfection of siRNA resulted in decreased expression of Notch1 and TGF-β (Fig 9). These findings suggest that the Notch1 and TGF-β signaling pathways might also be involved in the regulation of cell growth and migration of denatured dermis and fibroblasts by miR-23b.

## Discussion

In this study, it was demonstrated that miR-23b is dynamically expressed during the recovery of heat-denatured dermis and fibroblasts, and inhibition of miR-23b results in enhanced proliferation and migration of heat-denatured fibroblasts. Further mechanistic investigation found that miR-23 directly targets and regulates Smad3 expression in denatured dermis and heat-denatured fibroblasts. Accordingly, ectopic overexpression of Smad3 was found to promote the growth and migration of heat-denatured fibroblasts. In addition, downregulation of miR-23 could increase the expression of Notch1 and TGF-β. Our results indicate that the recovery of heat-denatured dermis and fibroblasts depends on the upregulation of Smad3, resulting from the downregulation of miR-23b.

The dermis is enriched with collagen and appendages and provides nourishment and support to the skin [14]. It has been well documented that the dermis plays an important role in burn wound healing and the amount of dermis remaining after excision of the eschar determines the quality of the scar [15,16]. Therefore, loss of dermis leads to a poor scar outcome after severe burns. First, epidermal appendages may also be removed so that the burn can no longer heal by re-epithelialization. Second, there will be less dermis to support the skin graft, resulting in increased contracture [17]. Fibroblasts are one of the major cell types that are required for the recovery of denatured dermis. However, the regulation of the growth and survival of fibroblasts in the dermis after severe burns remains largely unknown. Our results demonstrate that miR-23b is decreased in the early stages of the recovery of heat-denatured dermis and fibroblasts and inhibition of miR-23b results in enhanced proliferation and migration of heat-denatured fibroblasts. Thus, the downregulation of miR-23 contributes to the survival and proliferation of fibroblasts in the dermis after severe burns. However, the mechanism of the suppression of miR-23b in the dermis after burns remains to be determined.

Several studies suggest that miR-23b may function as either a tumor suppressor gene [18] or oncogene [19] and play important roles in cell proliferation, migration and differentiation [20–23], depending on the type of cancer. miR-23b has been reported to promote tolerogenic properties of dendritic cells via inhibition of the Notch1/NF-κB signaling pathways [24]. Moreover, miR-23b cluster miRNA was diminished during the termination of liver regeneration and regulated the growth and differentiation of liver stem cells through TGF-β/bone morphogenetic protein signaling [25,26]. In thyroid cells, miR-23b and miR-29b can promote cell growth by targeting Smad3 [27]. Li et al. found that miR-23b was significantly upregulated in keloid fibroblasts and may partially contribute to the etiology of keloids by affecting several signaling pathways [28]. TGF-β is a superfamily of cytokines and plays important roles in wound healing and tissue repair [29]. Bai et al. found that loureirin B could inhibit scar fibroblast proliferation through the TGF-β1/Smad2/3 pathway [30]. However, the molecular mechanism of the regulation of these various signal transduction pathways by miR-23b, especially Notch1/NF-κB and TGF-β/bone morphogenetic protein signaling pathways, remains elusive. In addition, our previous study showed that miR-23b is downregulated in the denatured dermis of deep burn patients [10]. However, the physiological consequence of miR-23b downregulation is largely unknown. In this study, it was found that miR-23 directly targets and regulates Smad3 expression in denatured dermis and heat-denatured fibroblasts. Furthermore, our results showed that ectopic overexpression of Smad3 promotes the growth and migration of heat-denatured fibroblasts, while silencing of Smad3 by siRNA results in decreased growth and migration of heat-denatured fibroblasts. In addition, Smad3 protein was upregulated with heat-denatured dermis, which was in agreement with the downregulation of miR-23b in denatured dermis. Our data demonstrates that targeting Smad3 is one of the mechanisms by which miR-23b controls the activity of many intracellular transduction pathways.

The TGF-β signaling pathway plays an important role in the regulation of cell growth, development and invasion in a variety of biological processes and the Notch signaling pathway is critical in the maintenance of tissue homeostasis by regulating cell proliferation and apoptosis. Moreover, both TGF-β and Notch signaling pathways are involved in the regulation of stem cell self-renewal and cell fate determination [31–33]. Our study showed that miR-23b downregulation resulting from transfection with inhibitor increased the levels of Notch1 and TGF-β. In addition, upregulation of Smad3 by pcDNA-Smad3 plasmid transfection in fibroblasts resulted in increased protein expression of Notch1 and TGF-β, whereas its downregulation by siRNA results in decreased protein expression of Notch1 and TGF-β. These findings suggest that miR-23b promotes the growth and migration of denatured dermis and fibroblasts by activing Notch1 and TGF-β signaling pathways.

In summary, our study demonstrates that miR-23b plays important roles in the regulation of the survival and growth of denatured dermis and suggests that miR-23b-mediated control of Smad3 expression may be fundamental for skin regeneration after severe burns.
